# Interception of Class III malocclusion with facemask therapy: A retrospective cohort study

**DOI:** 10.4317/jced.62798

**Published:** 2025-06-01

**Authors:** Jenny Angélica Saldarriaga-Valencia, Adriana Santamaria, Emery Álvarez-Varela, Yasmy Quintero, Ruben Darío Manrique-Hernández, Carlos M. Ardila, Ary dos Santos-Pinto

**Affiliations:** 1 Department of Morphology, Genetics, Orthodontics and Pediatric Dentistry, São Paulo State University (Unesp), Araraquara School of Dentistry, Araraquara, São Paulo, Brazil. Pediatric Dentistry, CES University, Medellín, Colombia; 2 Pediatric Dentistry and MSc of Dental Sciences, CES University, Medellín, Colombia; 3 Postgraduate in Pediatric Dentistry and MSc of Dental Sciences, CES University, Medellín, Colombia; 4 Pediatric Dentistry, CES University, Medellín, Colombia. Department of Morphology, Genetics, Orthodontics and Pediatric Dentistry, São Paulo State University (UNESP), Araraquara School of Dentistry, Araraquara, São Paulo, Brazil; 5 Pharmaceutical Chemistry. MSc & PhD in Epidemiology. CES University, Medellín, Colombia; 6 Department of Periodontics, Saveetha Dental College, Saveetha Institute of Medical and technology sciences, SIMATS, Saveetha. University, Chennai, Tamil Nadu, India; 7 Department of Basic Sciences, Biomedical Stomatology Research Group, Faculty of Dentistry, Universidad de Antioquia U de A, Medellín, Colombia; 8 Department of Morphology, Orthodontics and Pediatric Dentistry, School of Dentistry, São Paulo State University (UNESP), Araraquara, São Paulo, Brazil

## Abstract

**Background:**

Class III malocclusion, characterized by maxillary retrusion or mandibular prognathism, poses significant challenges in orthodontic treatment. Early intervention with facemask therapy aims to correct skeletal discrepancies, but outcomes vary. This study evaluates the efficacy of rapid palatal expansion (RPE) and facemask therapy in improving maxillomandibular relationships in growing children with Class III malocclusion.

**Material and Methods:**

A retrospective cohort study was conducted on 37 patients (mean age 7.5 ± 1.1 years) treated with RPE and Petit facemask therapy. Cephalometric radiographs were analyzed at treatment onset (T0) and completion (T1). Patients were divided into Group 1 (favorable outcome, ANB angle increase) and Group 2 (unfavorable outcome, ANB unchanged/decreased). Statistical analysis included dependent and independent t-tests to compare skeletal and dental changes.

**Results:**

Group 1 (62.2% of patients) showed significant improvements in ANB angle (1.85° increase), maxillary position (SNA, A-Nperp), and cranial base dimensions (S-N, S-Ar). Group 2 (37.8%) exhibited worsening maxillomandibular relationships (ANB decrease of 1.0°). Key differences included cranial base angle (NSAr), maxillary sagittal position, and mandibular prognathism (Pog-Nperp). Vertical growth increased in both groups, with no significant rotational changes in the mandible or maxilla.

**Conclusions:**

Facemask therapy with RPE effectively improved skeletal Class III malocclusion in 62.2% of cases, primarily through maxillary advancement and cranial base adaptation. Unfavorable outcomes were associated with larger initial ANB angles and mandibular prognathism. Early intervention during prepubertal growth stages is recommended for optimal results.

** Key words:**Class III malocclusion, Orthopedic fixation devices, Maxillary expansion, Cephalometry, Retrospective studies.

## Introduction

Class III Class III malocclusion is a growth-related dentofacial deformity (dysplasia) characterized by a bimaxillary sagittal discrepancy, along with transverse and vertical alterations. This dysplasia is defined by either maxillary retrusion (maxillary hypoplasia), excessive mandibular growth (mandibular prognathism), or a combination of both (1). The persistence of its unfavorable mandibular growth pattern relative to the maxilla significantly exacerbates Class III disharmony with continued growth (2).

The etiology of Class III malocclusion is multifactorial, resulting from an interaction of hereditary and environmental factors. A strong genetic influence is associated with deviations in normal craniofacial growth (1,2). Environmental factors, such as enlarged tonsils, oral breathing, congenital anatomic defects, hormonal alterations, mandibular protrusion, altered craniocervical posture, trauma, diseases, irregular eruption of permanent incisors, or premature loss of primary incisors, have also been implicated (1).

The prevalence of Class III skeletal malocclusion varies significantly among and within different racial, ethnic, and geographic populations. Its incidence in Caucasians is approximately 3–5% (3), whereas in Asians (Japanese and Chinese), it increases to 14% (2). In American populations, the incidence is approximately 5%, and among Latinos, it is about 9.1% (5). A study by Thilander *et al*. found a prevalence of 3.7% in a Colombian population of 4,724 children and adolescents (6).

The treatment of Class III malocclusion in growing children is one of the most challenging aspects of orthodontics. The unfavorable growth pattern in children with Class III discrepancy often requires early orthopedic intervention aimed at growth modification, including maxillary protraction, functional appliances, and chin cup therapy. The necessity of early treatment and interceptive care remains controversial, as outcomes are often partial and dependent on the individual’s future growth trajectory until maxillomandibular growth is complete (1,2).

Previous clinical studies have demonstrated that applying orthopedic forces to the craniofacial complex during early growth stages or the initial mixed dentition can improve Class III malocclusion (7,8). The most effective treatment reported during the mixed dentition is maxillary protraction combined with maxillary expansion (3,9,10). Maxillary expansion induces forward and downward maxillary movement by affecting the intermaxillary and circummaxillary sutures, enhancing the response to protraction forces from a facemask (11). This expansion can be achieved using a Hyrax-type intraoral appliance cemented to the upper molars, combined with maxillary protraction via a facemask anchored to the forehead and chin and connected to intraoral hooks (12).

The principle of maxillary protraction involves applying directed forces at an early age to weakly fused circummaxillary sutures to stimulate bone apposition. The facemask technique was first described over a century ago by Potpeschnigg in 1875. In 1976, Delaire introduced a facemask design for Class III treatment, later modified by Petit in 1983 to increase the applied force and reduce total treatment duration (13).

Clinical and cephalometric studies have shown effects of facemask therapy including anterior maxillary displacement (14,15), extrusion and mesialization of upper molars (16,17), downward and backward mandibular rotation (13-15), forward movement of maxillary teeth, labial inclination of upper incisors and lingual inclination of lower incisors (18), increased SNA angle (19), increased ANB angle, increased Witts angle (20), decreased SNB angle (8), anterior displacement of point A, retrusion of point B, increased facial angle (NaPg) (20), increased vertical dimension (18,19) and a constriction of the anterior palate (16).

Despite clinical success, post-treatment changes following maxillary protraction should be closely monitored due to the unpredicTable growth pattern of Class III patients and a tendency for relapse. Contributing factors include unsTable maxillary displacement, posterior mandibular rotation, unfavorable mandibular growth, and dental inclination changes (2).

The purpose of this study was therefore to evaluate the factors associated with favorable skeletal outcomes in intercepting developing Class III malocclusion during the mixed dentition using protraction facemask therapy.

## Material and Methods

This retrospective cohort study involved a longitudinal analysis and was conducted after obtaining prior approval from the CES University Institutional Human Research Ethics Committee. Written informed consent was obtained from both parents and patients.

The sample comprised 37 patients diagnosed with Class III malocclusion (mean age: 7.5 ± 1.1 years) who were consecutively treated with rapid palatal expansion (RPE) and protraction facemask (FM) therapy at three private pediatric dentistry clinics in Medellín, Colombia. The cohort included 20 males (mean age: 7.5 ± 1.2 years) and 17 females (mean age: 7.4 ± 1.0 years).

Selection Criteria included patients in the mixed dentition with Class III molar relationship, anterior crossbite or edge to edge incisal relationship, without prior dental extractions or history of maxillary orthopedic treatment. All patients were classified as CS1 or CS2 according to their cervical vertebral maturation stage. Individuals who presented syndromes, maxillary dental anomalies, premature tooth exfoliation in the maxillary arch, craniofacial anomalies (including cleft lip and palate), and Class III malocclusion due to mandibular prognathism were excluded from the study.

**Figure 1:**
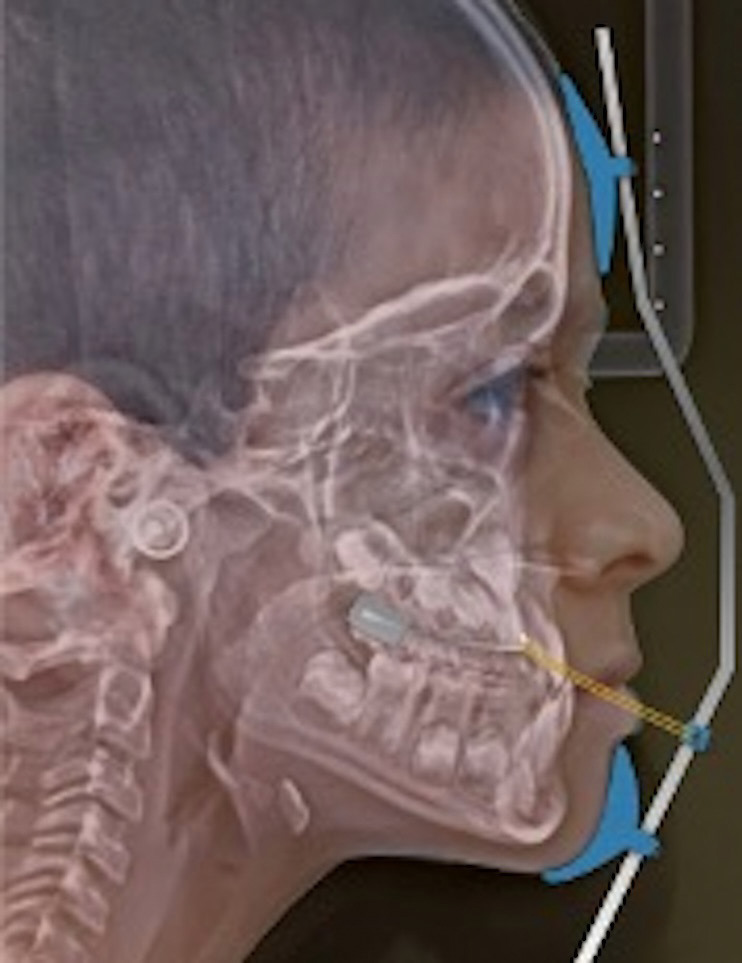
Facemask for maxillary protraction with elastics.

Cephalometric radiographs of patients who underwent a standardized treatment protocol of Class III skeletal malocclusion were selected. The protocol was similar to that described by Turley (17) in which rapid palatal expansion (RPE) was performed with a Hyrax-type screw supported on bands on the first upper permanent molars; the Hyrax expander was activated 0.25 mm/day during 20 days until reaching the appropriate transverse ratio depending on the initial condition of each patient. The Petit face mask was used for maxillary protraction during 14 to 16 hours/day, with elastics that generated 16 ounces force (300 to 500 grams) per side and with an anterior and lower force vector of 30 to 40 degrees with respect to the occlusal plane. Elastics were placed and extended from intra-oral hooks located at the canine level on both sides of the facemask to perform the maxillary protraction (Fig. 1). The facemask was used to correct the anterior crossbite and facial aesthetics. All patients achieved complete Class III correction with normal overjet, overbite, canine, and molar relationship.

Cephalometric radiographs were evaluated at the onset of treatment (T0) and at the end of treatment (T1). Mean treatment time was 1.6 ± 0.5 years. The sample design was non-probabilistic, given that records were selected by convenience of patients who had undergone successful treatment. Information gathered from the cephalometric records and medical history was entered into a database.An expert operator standardized the radiographic magnification factor and carried out tracings and measurements on radiographs with an intraclass correlation coefficient of 0.80, with high concordance between the expert operator and the author, as indicated by an intraclass correlation coefficient (U1SN: 0.99 and PmFh: 0.983).

- Measurements

All radiographs were traced and measured by the same operator, after a pilot test in which inter operator error was evaluated on 15 radiographs chosen randomly. Radiographs were standardized for magnification, to correct and guarantee a magnification of less than 10%.

**Figure 2:**
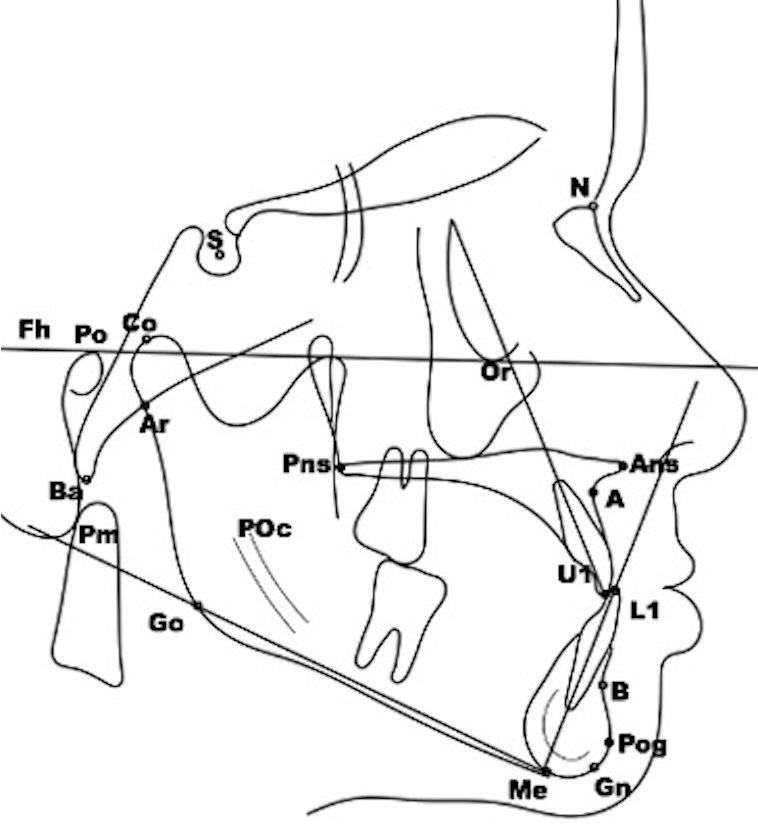
Anatomical points, lines and planes used for cephalometric analysis. *Pm=Mandibular plane (Go-Me), Fh=Frankfort horizontal plane (Porion-Orbitale), Pp= Palatal plane (Ans-Pns); Nperp= Nasion line perpendicular to the Fh; PFH= Posterior facial height (S-Go) and AFH= Anterior facial height (N-Me).

The cephalometric analysis (Fig. 2) included cranial base dimensions (S-N, S-Ar) and angulation (NSAr); maxillary position (SNA, A-Nperp,) and dimension (Co-A, Ans-Pns, Ba-A, Ba-Ans); mandibular position (SNB, Pog-Nperp), dimension (Co-Gn, Go-Gn) and angulation ArGoMe, PmFh); maxillomandibular angular relationship (ANB, DifMxMd, PpPm); Vertical dimension (N-Me, S-Go, N-Ans, Ans-Me, Ar-Go) and proportion (PFH/AFH); Upper Incisor angulation (U1SN, U1Fh) and position (U1-NA, U1-APog); Lower incisor angulation (L1Pm) and position (L1-NB, L1-APog); and angular interincisal relationship (U1L1).

- Study groups

For comparison purposes, the sample was divided into two groups based on the increase of the ANB angle after the treatment. Group 1 consisted of 23 patients with a mean age of 7.5 ± 1.2 years (nine males 7.4 ± 1.0 years old and fourteen females 7.6 ± 1.3 years old), who showed a favorable outcome, with an increase in the ANB angle after expansion and protraction.

Group 2 consisted of 14 patients (eight males 7.3 ± 1.1 years old and six females 7.4 ± 1.2 years old) who presented an unfavorable outcome, whose ANB angle remained unchanged or decreased after expansion and protraction. Group 1 represented 62.16%, while group 2 represented 37.84% of the sample.

- Statistical analysis

Prior to the analysis, a calibration process was carried out between the main author and an expert professional considered as the gold standard. A set of measurements of 15 subjects was used and intraclass correlation coefficient (ICC) was estimated. The author was calibrated when the ICC was greater than 0.9 for each measurement.

To verify normality of the data, the Shapiro-Wilk test was carried out. Mean and standard deviations were calculated for the variables with normal distribution. Median and confidence interval at 95% probability were calculated for the variables that did not exhibit normal distribution. The Levene test was applied to verify the homogeneity of variances between the groups.

The student’s t-test for dependent samples was used to compare the means before and after treatment ( Table 1, Table 2) and the student’s t-test for independent samples was used to compare treatment changes achieved between the two groups ( Table 3). Using the G*Power Version 3.1.9 software (Franz Faul, Germany) and considering the student’s t-test for differences between two independent means (n1=23 and n2=14) with an α error of 0.05 and an effect size of 0.88 the calculated power was 0.80 (1-β err prob).

The level of significance adopted for rejection of the hypotheses was 0.05. The statistical analysis was performed with the SPSS program - version 19.0.

## Results

Data displayed on  Table 1 indicates that there was no significant difference between groups 1 and 2 at the onset of treatment (T0), and significant differences were observed for the ANB angle, which was 1.4 ± 2.2 for group 1 and 3.3 3.3 ± 2.2 for group 2.

After treatment (T1), the ANB of both groups did not differ and the only significant difference was found in the NSAr variable which was higher in group 2 with an average value of 127.5 ± 6.0 degrees in relation to group 1 that presented an average value of 123.0 ± 6.7 degrees ( Table 2).

Table 3 shows the significant differences between the groups at the cranial base represented by NSAr, in maxillomandibular sagittal relationship (SNA), with maxillary dimensions measured with A-NPerp and mandibular ones with Pog-NPerp, that influenced the DifMxMd and NApgSN_Me measurement, and dental changes represented by variations in U1-APog and APogP1Po.

The results displayed on  Table 3 indicate that the differences between groups were related to:

1. Cranial base differences. In group 1 there was a significant increase in the anterior (S-N) and posterior (S-Ar) dimensions of the cranial base, while none was evident in group 2. Posterior cranial length increased 1 mm in group 1 and 0.5 mm in group 2. The most significant difference between groups was the NSAr angle which showed a decrease of 1 degree closing pattern in group 1 and an increase of 0.7 degree opening pattern in group 2.

2. Maxillary position contributed to the differences between two groups, with statistical differences observed between SNA and A-Nper. In Group 1, both measures showed a positive increase indicating a forward movement of the maxilla while group 2 showed a negative increase, indicating that maxilla did not follow the anterior growth movement of its counterpart. Although there were no differences in maxillary length (Ans-Pns) between groups, there was a significant increase in the Ans-Pns of 1,5 mm in group 1 and only 0.5 mm in group 2. Ba-A and Ba-Ans lengths showed a significant increase in both groups and combined with the difference observed in the cranial angle (NSAr) explain the most anterior position of the maxilla in group 1.

3. The mandibular position also contributed with Pog-Nperp showing statistical differences between groups. Although the outcome was favorable, in group 1 there was an increase in the mandibular prognathism by 1.6 mm ( Table 3) with an initial value of Pog-Nper of -0.8 mm and a final value of -2.4 mm. On the contrary, an unfavorable outcome was observed in group 2, with Pog-Nperp decreasing 0.7 mm, from an initial value of -3.3 mm to -2.6 mm. Variations of Pog-Nperp within the groups was not significant (Table 1, 2 and 3). Measurements of mandibular dimension (Co-Gn, Go-Gn) showed significant increase in both groups but with no statistical difference between them, whereas the mean values were greater in group 1 than in group 2. Co-Gn and Go-Gn increased 1.4 mm (3.1 mm and 4.5 mm) and 1.1 mm (2.5 mm and 3.4 mm) less in group 1 than in group 2.

4. The maxillomandibular relationship (ANB) increased 1.8 degrees in group 1, while it decreased 1.0 mm in group 2. The maxillomandibular difference did not change significantly in group 1 (DifMxMd) while in group 2 there was a significant increase of 3.3 mm indicating a worsening of the relationship. In group 2, Co-A increased 1.2 mm which was not significant and Co-Gn increased 4.5 mm, which was significant. Moreover, in group 1, Co-A significantly increased 2.5 mm and Co-Gn only 3.1 mm.

There were no significant changes in the inclination of the palatal plane (PpPm) and mandibular plane (PmFh) between T0 and T1, except for PpPm, which significantly increased 1.5 degree in group 1, while no significant differences for PmFh and PpPm between groups were observed ( Table 1, Table 2, Table 3).

5. Both groups evidenced a significant increase in vertical dimension, but with no statistical difference between them. However, mean values for N-Me and S-Go were greater in group 2 than in group 1 and changes in S-Go were smaller than in N-Me which led to a greater increase for group 1 PmFh (1 degree, from 28.1 to 29.1 degree) but not statistically different from group 2 (0.1 degree, from 29.3 to 29.4 degree). A significant decrease in the PFH/AFH angle in both groups was also observed. Variation in PFH/AFH was greater in group 1 (-4.9% from 89.8% to 84.9%) than in group 2 (-3.8% from 89.0% to 85.2%) but not statistically significant.

6. Dental differences: There were no differences in the upper incisor position and inclination between groups. Both groups showed significant increase in upper incisor inclination (U1SN and U1Fh) and in upper incisor position (U1-NA and U1-APog). The lower incisor was significantly positioned posteriorly in group 1 approximately 1 mm (L1-APog) while in group 2 there was minimal variation in lower incisor position. Moreover, protrusion of the lower incisor with relation to the NB line did not vary with treatment. The inclination of the lower incisor (L1-Pm) did not vary significantly with treatment in both groups (L1-Pm and U1L1).

## Discussion

The evaluation of maxillary expansion and protraction efficacy in Class III malocclusion treatment focuses on two fundamental aspects: correction of skeletal disharmony and dentoalveolar discrepancy. In this study, dentoalveolar correction effectiveness was demonstrated by complete resolution of Class III dental relationships and anterior/posterior crossbites in all patients, consistent with previous findings (21). However, controversy remains regarding its effectiveness in improving skeletal discrepancies.

Maxillary expansion induces forward and downward maxillary movement through its effect on intermaxillary and circummaxillary sutures, enhancing the response to facial mask protraction forces (11). The principle of maxillary protraction involves applying directed forces to weakly fused circummaxillary sutures in young patients, stimulating bone apposition at these sites (13). Expansion objectives include posterior crossbite correction and maxillary disarticulation. Both facemask therapy with and without rapid maxillary expansion (RME) improve skeletal Class III malocclusion, suggesting RME use should be based on clinical criteria rather than being mandatory for Class III correction (13). The primary goal of early orthodontic treatment is to establish physiological occlusal conditions favoring proper dentofacial development.

In this study, 37 Class III patients divided in one group of 23 and another of 14 patients treated with Hyrax maxillary expansion followed by maxillary protraction face mask therapy showed improvement in their maxillomandibular relation measured by the ANB angle. Based on the results, 62,6 % of the cases showed a mean of 1.4 mm improvement in the Class III skeletal disharmony while 37,8% presented worsening with a mean of 3.3 mm. These results are in agreement with those of Mandall *et al*. who reported a success rate of 70% in a multicentric randomized controlled trial (22) and concluded that early orthopedic Class III treatment with facemask protraction in patients under 10 years of age, was skeletally and dentally effective and did not result in TMJ dysfunction and conferred a clinically significant psychosocial benefit. Masucci *et al*. (23) evidenced that in the long term, rapid maxillary expansion and facemask therapy led to successful outcomes in approximately 73% of Class III patients and favorable skeletal changes were mainly due to significant improvements in mandibular sagittal position.

These results are also in accordance with Kajiyama *et al*. (24) who reported 70% skeletal movement and 30% compensation in the inclinations of anterior teeth in the horizontal correction of the anterior crossbites in Class III patients in the mixed dentition treated with maxillary protraction. This study was carried out to understand and establish the contributing factors to these results.

Our results indicated that the severity of the skeletal Class III before treatment measured by the ANB angle had a negative correlation with changes in ANB with treatment (Pearson coefficient of correlation r = -0.4). The lower the ANB at the onset of treatment the greater the increase in ANB with treatment (Group 1). This result indicated that the group with the lowest ANB angles responded more favorably to the maxillary protraction, increasing its value in 1.85 degrees ( Table 3). In group 2 which presented an unfavorable outcome, initial ANB angle was greater than in group 1 ( Table 1) and showed a reduction of 1.0 degree ( Table 3), indicating a worsening in maxillomandibular discrepancy despite treatment. Gu (15) indicated that severe maxillomandibular discrepancy, increased vertical dimension and a prognathic mandible were unfavorable factors for long-term stability following early treatment of severe Class III subjects with facemask protraction. Hence, we could expect that individuals in group 2 with unfavorable outcomes, were more susceptible to long term instability (ANB was 3.3 ± 2.2 at T0 and 2.3 ± 2.2 at T1). In group 1 with favorable outcomes, initial ANB was worse, but with treatment it increased and presented a better outcome and more favorable long-term stability (ANB 1.3 ± 2.2 at T0 and 3.2 ± 2.3 at T1).

The overall correlation between variations ANB and SNA was r =0.63 (Pearson’s coefficient of correlation) indicating that protraction was important factor in achieving a favorable outcome. A-Nperp had an r = 0.43 and Pog-Nperp an r = -0.47 with changes in ANB with treatment. Significant differences in variations of the SNA angle could be attributed to a significant negative evolution of this angle in group1 (-0.8 degrees) and to a small positive and non- significant evolution in group 2 (1.1 degrees). SNB did not differ between groups and did not present a significant evolution (-0.7 degree in group 1 and 0.2 degree in group 2) ( Table 3).

Many studies have shown that most Class III patients that present maxillary deficiency as well as mandibular excess could benefit from early orthopedic mechanics applied to protract maxillary structures (19-21). Our results point to the same direction, although no significant differences in maxillary measurements between groups were observed; in group 1 a significant increase in maxillary length (Co-A and Ans-Pns) as result of treatment was evident. In group 1 the favorable outcome was related to the most anterior positioning of the Maxilla (A-Nperp 0.7 mm) and in group 2 the unfavorable outcome was related to a more posterior positioning of the Maxilla (A-Nperp -0.5 mm). The combination of a reciprocal retractive force with the face mask therapy provided a favorable outcome with skeletal and dental changes in the maxilla and mandible. Our results indicated that in group 1, the favorable outcome was related to a posterior positioning of the Mandible (Pog-Nperp -1.6 mm) and in group 2 the unfavorable outcome was due to an anterior positioning of mandible (Pog-Nperp 0.7 mm). Data indicates that mandible length increased significantly (Co-Go and Go-Gn) in both groups but slightly more in group 2 than group 1. In this way the combination increased maxillary growth and decreased mandibular growth resulted in a non-significant increase in maxillomandibular difference in group 1 (DifMxMd of 0.6 mm) and a significant increase in group 2 (DifMxMd of 3.3 mm) contributing significantly to the difference between groups.

Most studies (9,20,24) that have assessed the results of maxillary expansion and protraction indicate that the maxilla moved forward (Ans and point A) but that when traction was applied on the first molars or if forces were parallel to the occlusal plane, the maxilla presents an anterior rotation evidenced by an increase in the palatal plane angulation (15). This rotation is an undesirable side effect that leads to an open bite tendency. In order to avoid this adverse occurrence, some authors (26) advocate applying the protraction force at the level of the canines and a 20 to 30 degree downward direction in relation to the occlusal plane. In this study, the Petit face mask was used for maxillary protraction observing three parameters which included the use of 16 ounce elastics for delivery of the force per side, facemask use of 14 to 16 hours per day, and application of forces as close as possible to center of resistance of the maxilla achieved by the inserting the elastics in hooks located at the level of canines and extended to the Petit-type face mask with an anterior and lower force vector of 30 to 40 degrees with respect to the occlusal plane (force direction). Said parameters were in agreement those proposed Yepes *et al*. in a systematic review (26). Our results are also in agreement with those of Keles *et al*. (27) that concluded that varying the force direction of maxillary protraction near the center of resistance of maxilla was an effective method to prevent undesirable side effects such as anterior rotation of the maxilla.

Our results indicated that the mandible did not present significant rotation with treatment, and that there were no significant differences in mandibular plane angle (PmFh) and the palatal/mandibular plane angle (PpPm) at the beginning and end of treatment indicating no significant maxillary or mandibular rotation ( Table 1, Table 2). Also,  Table 3 indicates that there were no differences in PmFh and PpPm as result of treatment. Salazar *et al*. (28) evaluated the vertical effect of face mask therapy on the growth pattern of the mandible and concluded that there was growth direction was sTable during treatment.

Vertical facial growth increased significantly during treatment evidenced by significant changes observed in anterior and posterior facial height. Although no significant changes occurred between groups, a greater increase of this dimension was evident in group 2. Table 3 shows greater values for N-Me, S-Go, Ar-Go and Ans-Me in group 2. The PFH/AFH decreased significantly in both groups without significant differences observed between them, indicating that there was greater increase in anterior facial height in relation to posterior facial height. According to Kwak *et al*. (29), vertical skeletal changes as a result of facemask therapy are significantly associated with severity of skeletal Class III malocclusion and mandibular plane angulation before treatment and the amount of forward maxillary growth during treatment and retention periods.

The important role of vertical skeletal relationships and the degree of cranial base flexure in the diagnostic and prognostic evaluation of Class III patients warrants analysis. Orthopedic treatment of Class III malocclusion might be unfavorable over the long term when a patient’s cephalometric records show an increased posterior facial height, an acute cranial base angle, and a steep mandibular plane angle at the onset of treatment (24). In addition, growing patients with forward position of the mandible, short ramus, increased mandibular length, and an obtuse gonial angle are highly associated with unfavorable treatment outcomes after pubertal growth (13-15). In our study, group 2 had a greater cranial base angle than in group 1, and cranial base angle modification with growth during treatment showed significant differences between groups. In group 1 there was 1.0 degree decrease and, while in group 2 there was an increase of 0.7 degrees.

These variations, although not significant, were the result of significant elongation of the cranial base in group 1. While S-N and S-Ar measures increased 1.0 mm in group 1, the posterior cranial base length increased 0.5 mm in group 2.

The prognosis of orthopedic treatment of skeletal Class III malocclusion is favorable when treatment is initiated before the pubertal growth peak. If left untreated, Class III malocclusion may worsen due to the adverse growth pattern resulting from the skeletal imbalance and unfavorable dental compensations (2). Consequently, early treatment is recommended for skeletal Class III malocclusion to obtain a balanced skeletal relationship, avoiding or minimizing the need of further complex orthodontic treatment and interventions such as orthognathic surgery (17-20).

According to the study by Franchi, Baccetti & McNamara (10), orthopedic treatment of Class III malocclusion was more effective when it was initiated at an early developmental phase (early mixed or late mixed dentition) rather than during later stages with respect to untreated Class III control groups. Patients treated with rapid maxillary expansion and facemask therapy in the late mixed dentition, however, still benefited from the treatment, but to a lesser degree. Early treatment produced significant favorable post-pubertal modifications in both maxillary and mandibular structures, whereas late treatment induced only a significant restriction of mandibular growth. Significant changes in mandibular size were associated with significant changes in mandibular shape only in early treated subjects. The main contribution to overall occlusal correction was related to skeletal modifications rather than dento-alveolar changes in both early and late treated groups. In our study, mandibular growth was significant and produced changes in the mandibular shape in both groups without significant differences observed between them, and also presented a closing effect in the angle between the ramus and corpus (gonial angle) of 1.7 degrees in group 1 and 1.4 degrees in group 2. Tahmina *et al*. (30) reported that a large gonial angle was present in the initial stage of the group that showed unsTable treatment results and that it increased over time. They also observed significant forward growth of the mandible, as well as a clockwise rotation and a counterclockwise rotation after treatment.

Early orthopedic facemask therapy improves skeletal relationships, which in turn minimizes excessive dental compensation such as overclosure of the mandible and lingual inclination of lower incisors (24). In this regard our findings indicate that dental movement followed skeletal growth pattern but did not differ significantly between groups. An increase in upper incisor angulation (U1SN and U1Fh) as well as protrusion (U1-NA and U1-APog) was observed.

Differences between groups were observed when protrusion was measured by the A-Pog line and as an effect the maxillo-mandibular changes. Lower incisors did not have a significant decrease in angulation (L1Pm) and no changes in protrusion (L1-NB) were evident. Protrusion of lower incisors measured by the A-Pog line also showed differences between groups with a significant decrease in group 1 also reflecting changes in the maxilla and mandible.

Class III malocclusion with an anterior crossbite is often accompanied by a functional shift. Early orthopedic treatment may help in eliminating centric occlusion/centric relation (CO/CR) discrepancies and avoid adverse growth reducing the complexity of subsequent comprehensive treatment. In mild and moderate Class III patients, early orthodontic or orthopedic treatment eliminate the necessity for orthognathic surgery in the adult age (27-30). Early correction of the transverse dimension and maximizing the growth potential of the maxilla may minimize the extent of the surgical procedures.

According to Zere *et al*. (1), the proper timing of interventions may rely on chronological age and phases of dentition for very young patients, and on other radiological indicators, such as cervical vertebral maturation and/or hand and wrist maturation methods for older children. The main goals of early intervention are to create a more favorable environment for growth and to improve the occlusal relationship: for example, correcting the crossbite and facial esthetics. Hence, interceptive treatment of Class III malocclusions should be undertaken if it prevents damage to the oral tissues and prevents or significantly reduces the amount, or severity, of future orthodontic and surgical intervention.

According to Franchi, Baccetti & McNamara (10) more effective craniofacial changes were obtained in patients treated with RME/FM in the early mixed dentition than the late mixed dentition. They also pointed that when the skeletal age is considered, obtained by the hand-and-wrist method, no difference was found in the effects of maxillary advancement after maxillary protraction comparing prepubertal with pubertal growth-peak groups, whereas the results were less effective in the post pubertal growth-peak.

Patients treated with rapid maxillary expansion and facemask therapy in the late mixed dentition, however, still benefited from the treatment, but to a lesser degree. Early treatment produced significant favorable post pubertal modifications in both maxillary and mandibular structures, whereas late treatment induced only a significant restriction of mandibular growth. Significant changes in mandibular size were associated with significant changes in mandibular shape only in early treated subjects. The main contribution to overall occlusal correction was related to skeletal modifications rather than dental changes in both early and late treated groups (22).

There were no age differences between patients in the groups of this study and all of them were in the mixed dentition phase and in vertebral maturation stage of CS1 and CS2 indicating that all were in the prepubertal growth phase. The findings corroborated that orthopedic treatment of Class III malocclusion was effective when it was initiated at an early developmental phase of the dentition.

## Conclusions

Class III malocclusion treated with Hyrax maxillary expansion followed by maxillary protraction with facemask therapy reduces Class III skeletal disharmony and improves the maxillomandibular relationship in 62,6 % of cases (ANB increase 1.85 ± 0.2º). In 37,8% of the cases there are a worsening in this relationship (ANB decrease 1,0 ± 0.2º). This outcome was influenced by significant differences that occurred at the cranial base (NSAr), the maxillary sagittal position (SNA and A-NPerp), the mandibular sagittal position (Pog-NPerp) and dento-alveolar changes (U1-APog and L1-Apog).

## Figures and Tables

**Table 1:** Descriptive statistics. Initial cephalometric values (t0) according to the group and t test for inter-group differences.

			Group 1			Group 2			Difference		Conf. Interval		Levene Test		t test	
Evaluation		Variables	n	Mean	S.D.	n	Mean	S.D.	Mean	E.P.	Lower	Upper	F	Sig.	t	P
		Age	23	7.50	1.19	14	7.38	1.09	0.12	0.39	-0.68	0.91	0.110	0.742	0.299	0.767
Cranial Base		S-N	23	65.64	3.59	14	64.86	3.83	0.78	1.25	-1.75	3.31	0.102	0.751	0.625	0.536
		S-Ar	23	29.45	2.98	14	30.59	2.30	-1.15	0.93	-3.04	0.75	1.038	0.315	-1.229	0.227
		NSAr	23	123.99	6.40	14	126.89	5.17	-2.90	2.02	-7.01	1.21	0.727	0.399	-1.433	0.161
Maxilla		SNA	23	80.11	4.10	14	79.96	4.15	0.15	1.40	-2.69	2.99	0.000	0.987	0.108	0.914
		A-Nperp	23	0.89	2.73	14	1.08	2.72	-0.19	0.92	-2.07	1.68	0.138	0.713	-0.207	0.838
		Co-A	23	79.53	3.76	14	81.11	5.31	-1.58	1.49	-4.61	1.45	1.200	0.281	-1.057	0.298
		Ans-Pns	23	48.21	2.35	14	50.21	3.56	-1.99	1.07	-4.23	0.24	4.406	0.043	-1.863	0.077
		Ba-A	23	85.73	4.50	14	86.72	5.78	-0.99	1.70	-4.44	2.46	0.877	0.355	-0.583	0.564
		Ba-Ans	23	89.13	4.28	14	90.57	6.27	-1.45	1.73	-4.96	2.07	2.496	0.123	-0.834	0.410
Mandible		SNB	23	78.75	3.67	14	76.67	3.74	2.08	1.25	-0.47	4.62	0.010	0.923	1.657	0.107
		Pog-Nperp	23	-0.77	4.90	14	-3.32	4.25	2.55	1.58	-0.66	5.77	0.003	0.956	1.612	0.116
		Co-Gn	23	105.41	5.25	14	105.76	7.73	-0.36	2.13	-4.68	3.97	2.403	0.130	-0.167	0.868
		Go-Gn	23	72.52	3.18	14	73.16	5.97	-0.65	1.73	-4.28	2.99	4.541	0.040	-0.375	0.712
		ArGoMe	23	129.57	4.04	14	128.94	2.81	0.63	1.23	-1.87	3.13	3.690	0.063	0.511	0.612
		PmFh	23	28.05	3.13	14	29.29	3.21	-1.24	1.07	-3.41	0.93	0.001	0.972	-1.157	0.255
Maxillomandibular relationship		ANB	23	1.36	2.22	14	3.29	2.20	-1.92	0.75	-3.44	-0.41	0.599	0.444	-2.572	0.015
		DiMxMd	23	25.84	3.29	14	24.61	4.54	1.23	1.29	-1.39	3.84	2.315	0.137	0.953	0.347
		PpPm	23	29.73	3.28	14	31.59	3.09	-1.86	1.09	-4.06	0.35	0.000	0.999	-1.705	0.097
Vertical		N-Me	23	106.55	5.65	14	108.62	8.09	-2.07	2.46	-7.20	3.05	5.552	0.024	-0.842	0.409
		S-Go	23	62.59	5.00	14	62.96	4.53	-0.37	1.64	-3.69	2.96	0.181	0.673	-0.224	0.824
		PFH/AFH	23	89.76	5.05	14	89.00	6.00	0.76	1.84	-2.97	4.49	1.807	0.187	0.414	0.681
		N-Ans	23	48.29	3.36	14	49.21	4.13	-0.92	1.24	-3.45	1.60	0.909	0.347	-0.743	0.462
		Ans-Me	23	59.78	3.71	14	61.61	5.08	-1.82	1.45	-4.76	1.11	4.070	0.051	-1.260	0.216
		Ar-Go	23	36.10	3.46	14	35.47	3.72	0.63	1.21	-1.81	3.08	0.201	0.657	0.525	0.603
Dental	Upper incisor	U1SN	23	99.47	6.25	14	90.48	27.24	8.99	7.40	-6.89	24.87	5.000	0.032	1.215	0.245
		U1Fh	23	110.31	5.72	14	100.92	29.95	9.39	8.09	-8.02	26.80	5.504	0.025	1.160	0.266
		U1-NA	23	1.59	1.66	14	1.17	2.21	0.42	0.64	-0.88	1.71	1.616	0.212	0.650	0.520
		U1-APog	23	2.71	2.15	14	3.28	2.07	-0.57	0.72	-2.03	0.89	0.071	0.791	-0.786	0.437
	Lower incisor	L1Pm	23	85.47	6.72	14	86.76	5.21	-1.30	2.10	-5.57	2.97	0.908	0.347	-0.618	0.540
		L1-NB	23	4.06	2.23	14	4.31	1.69	-0.25	0.69	-1.66	1.16	1.483	0.231	-0.361	0.720
		L1-Apog	23	3.47	2.26	14	2.47	2.06	1.00	0.74	-0.51	2.50	0.471	0.497	1.346	0.187
	Interincisal	U1L1	23	136.00	8.58	14	125.54	37.43	10.47	8.07	-5.91	26.85	3.412	0.073	1.297	0.203

**Table 2:** Descriptive statistics. Final cephalometric values (t1) according to the group and t test for inter-group differences.

			Group 1			Group 2			Difference		Conf. Interval		Levene Test		t test	
Evaluation		Variables	n	Mean	S.D.	n	Mean	S.D.	Mean	E.P.	Lower	Upper	F	Sig.	t	p
		Age	23	9.12	1.23	14	8.83	0.88	0.29	0.38	-0.48	1.06	1.094	0.303	0.767	0.448
Cranial Base		S-N	23	66.73	3.26	14	66.10	3.43	0.63	1.13	-1.66	2.91	0.393	0.535	0.556	0.582
		S-Ar	23	30.47	2.90	14	31.08	2.14	-0.61	0.90	-2.43	1.21	1.728	0.197	-0.684	0.499
		NSAr	23	122.97	6.75	14	127.54	6.03	-4.57	2.20	-9.04	-0.11	0.136	0.714	-2.079	0.045
Maxilla		SNA	23	81.26	4.91	14	79.16	4.73	2.10	1.64	-1.23	5.44	0.155	0.696	1.281	0.209
		A-Nperp	23	1.59	2.76	14	0.62	3.58	0.97	1.05	-1.16	3.10	0.136	0.714	0.927	0.361
		Co-A	23	82.02	3.60	14	82.34	4.50	-0.32	1.34	-3.04	2.40	1.148	0.291	-0.239	0.812
		Ans-Pns	23	49.73	2.41	14	50.73	3.06	-1.00	0.90	-2.84	0.83	1.341	0.255	-1.108	0.275
		Ba-A	23	88.25	4.31	14	88.76	5.16	-0.52	1.57	-3.71	2.68	0.157	0.694	-0.328	0.745
		Ba-Ans	23	91.59	4.28	14	92.71	5.42	-1.12	1.61	-4.38	2.14	0.457	0.503	-0.698	0.490
Mandible		SNB	23	78.05	4.31	14	76.89	4.21	1.17	1.45	-1.78	4.11	0.119	0.733	0.805	0.426
		Pog-Nperp	23	-2.42	4.77	14	-2.59	5.53	0.17	1.72	-3.32	3.66	0.994	0.326	0.098	0.922
		Co-Gn	23	108.49	4.95	14	110.24	7.24	-1.75	2.00	-5.81	2.31	1.015	0.321	-0.876	0.387
		Go-Gn	23	75.06	3.94	14	76.61	5.79	-1.55	1.60	-4.79	1.70	0.407	0.528	-0.968	0.340
		ArGoMe	23	127.89	3.85	14	127.54	3.38	0.35	1.25	-2.18	2.88	0.458	0.503	0.282	0.780
		PmFh	23	29.14	3.48	14	29.43	4.22	-0.29	1.28	-2.89	2.30	0.216	0.645	-0.230	0.820
Maxillomandibular relationship		ANB	23	3.21	2.32	14	2.27	2.22	0.94	0.78	-0.64	2.51	2.213	0.146	1.209	0.235
		Dif MxMd	23	26.44	3.81	14	27.88	5.14	-1.44	1.48	-4.43	1.56	0.906	0.348	-0.976	0.336
		PpPm	23	31.27	3.95	14	31.99	4.18	-0.73	1.37	-3.51	2.05	0.288	0.595	-0.531	0.599
Vertical		N-Me	23	108.43	14.49	14	113.92	7.58	-5.50	4.20	-14.02	3.03	0.112	0.740	-1.309	0.199
		S-Go	23	64.76	4.34	14	66.10	3.69	-1.34	1.39	-4.17	1.48	0.094	0.761	-0.965	0.341
		PFH/AFH	23	84.86	8.72	14	85.24	6.00	-0.38	2.65	-5.76	5.00	0.449	0.507	-0.144	0.886
		N-Ans	23	50.24	3.11	14	51.79	3.57	-1.55	1.11	-3.81	0.72	0.142	0.709	-1.387	0.174
		Ans-Me	23	62.57	4.38	14	63.96	5.07	-1.40	1.58	-4.60	1.80	0.660	0.422	-0.888	0.381
		Ar-Go	23	36.77	2.87	14	38.06	3.87	-1.29	1.11	-3.55	0.96	0.894	0.351	-1.163	0.253
Dental	Upper incisor	U1SN	23	103.47	7.20	14	95.01	27.91	8.46	6.08	-3.89	20.80	2.823	0.102	1.391	0.173
		U1Fh	23	113.94	6.00	14	105.82	30.82	8.12	6.57	-5.22	21.45	3.563	0.067	1.236	0.225
		U1-NA	23	3.24	2.19	14	3.51	2.43	-0.26	0.77	-1.83	1.31	0.158	0.693	-0.338	0.737
		U1-APog	23	5.76	2.68	14	5.06	2.59	0.70	0.90	-1.13	2.52	0.052	0.821	0.775	0.443
	Lower incisor	L1Pm	23	83.89	7.03	14	85.41	5.05	-1.52	2.16	-5.90	2.87	2.401	0.130	-0.702	0.487
		L1-NB	23	4.27	2.51	14	4.49	1.70	-0.21	0.76	-1.75	1.33	2.168	0.150	-0.279	0.782
		L1-Apog	23	2.42	2.48	14	3.01	1.98	-0.59	0.78	-2.17	1.00	1.997	0.166	-0.751	0.458
	Interincisal	U1L1	23	132.85	9.12	14	121.10	35.70	11.75	7.77	-4.03	27.53	2.559	0.119	1.512	0.140

**Table 3:** Descriptive statistics. Cephalometric values of the treatment effects (t1-t0) accordig to the group and t test for intergroup differences.

			Group 1			Group 2			Difference		Conf. Interval		Levene Test		t test	
Evaluation		Variables	Mean	S.E.	Mean	S.E.	Mean	E.P.	Lower	Upper	F	Sig.	t	p
		Age	1.62	**	0.12	1.45	**	0.10	0.17	0.17	-0.17	0.52	2.872	0.099	1.024	0.313
Cranial Base		S-N	1.08	**	0.47	1.24	ns	0.61	-0.15	0.77	-1.72	1.41	0.000	0.985	-0.199	0.843
		S-Ar	1.02	**	0.33	0.49	ns	0.47	0.53	0.55	-0.59	1.66	0.811	0.374	0.960	0.344
		NSAr	-1.02	ns	0.54	0.66	ns	0.54	-1.68	0.82	-3.33	-0.02	0.540	0.468	-2.049	0.048
Maxilla		SNA	1.15	ns	0.44	-0.80	*	0.36	1.95	0.63	0.68	3.23	0.848	0.363	3.107	0.004
		A-Nperp	0.70	ns	0.30	-0.46	ns	0.46	1.16	0.52	0.10	2.22	0.132	0.718	2.221	0.033
		Co-A	2.49	**	0.45	1.24	ns	0.87	1.26	0.89	-0.55	3.06	1.845	0.183	1.411	0.167
		Ans-Pns	1.51	**	0.37	0.52	ns	0.51	0.99	0.62	-0.26	2.25	0.487	0.490	1.604	0.118
		Ba-A	2.52	**	0.54	2.04	*	0.70	0.47	0.88	-1.32	2.27	0.084	0.774	0.537	0.595
		Ba-Ans	2.46	**	0.46	2.14	*	0.77	0.33	0.84	-1.38	2.03	0.423	0.519	0.386	0.702
Mandible		SNB	-0.70	ns	0.39	0.21	ns	0.29	-0.91	0.55	-2.02	0.20	1.962	0.170	-1.667	0.104
		Pog-Nperp	-1.65	ns	0.68	0.74	ns	0.80	-2.38	1.07	-4.55	-0.22	0.013	0.909	-2.234	0.032
		Co-Gn	3.08	**	0.82	4.48	**	1.15	-1.40	1.39	-4.21	1.42	0.015	0.902	-1.007	0.321
		Go-Gn	2.54	**	0.60	3.44	**	1.06	-0.90	1.13	-3.19	1.39	1.435	0.239	-0.796	0.431
		ArGoMe	-1.68	**	0.35	-1.40	**	0.38	-0.28	0.54	-1.38	0.82	0.793	0.379	-0.515	0.610
		PmFh	1.09	ns	0.50	0.14	ns	0.51	0.94	0.76	-0.59	2.48	0.855	0.362	1.247	0.221
Maxillomandibular relationship		ANB	1.85	**	0.20	-1.01	**	0.22	2.86	0.30	2.24	3.48	0.001	0.978	9.397	0.000
		DifMxMd	0.60	ns	0.45	3.27	**	0.45	-2.67	0.67	-4.04	-1.30	0.618	0.437	-3.954	0.000
		PpPm	1.54	*	0.45	0.41	ns	0.81	1.13	0.85	-0.60	2.85	0.211	0.649	1.326	0.193
Vertical		N-Me	1.88	**	2.71	5.30	**	1.02	-3.42	3.58	-10.69	3.84	1.087	0.304	-0.956	0.345
		S-Go	2.17	**	0.72	3.14	**	0.53	-0.98	1.02	-3.04	1.09	2.461	0.126	-0.962	0.343
		PFH/AFH	-4.90	**	1.46	-3.76	**	0.79	-1.14	1.98	-5.17	2.88	2.688	0.110	-0.576	0.568
		N-Ans	1.95	**	0.53	2.57	**	0.49	-0.62	0.79	-2.22	0.97	1.128	0.295	-0.794	0.432
		Ans-Me	2.78	**	0.43	2.36	*	0.84	0.43	0.85	-1.30	2.15	2.180	0.149	0.500	0.620
		Ar-Go	0.66	ns	0.68	2.59	**	0.57	-1.92	0.98	-3.91	0.06	1.341	0.255	-1.971	0.057
Dental	Upper incisor	U1SN	4.00	*	1.53	4.53	*	1.54	-0.53	2.31	-5.22	4.15	1.036	0.316	-0.231	0.819
		U1Fh	3.63	*	1.49	4.90	**	1.46	-1.27	2.22	-5.79	3.25	1.366	0.250	-0.571	0.572
		U1-NA	1.66	**	0.39	2.34	**	0.44	-0.68	0.60	-1.90	0.54	0.384	0.539	-1.128	0.267
		U1-APog	3.05	**	0.35	1.79	**	0.41	1.26	0.55	0.14	2.38	0.014	0.905	2.290	0.028
	Lower incisor	L1Pm	-1.57	ns	1.11	-1.36	ns	1.15	-0.22	1.69	-3.64	3.21	1.506	0.228	-0.128	0.899
		L1-NB	0.22	ns	0.28	0.18	ns	0.30	0.04	0.43	-0.84	0.91	0.389	0.537	0.090	0.929
		L1-Apog	-1.05	**	0.29	0.54	ns	0.34	-1.58	0.46	-2.51	-0.66	0.272	0.605	-3.463	0.001
	Interincisal	U1L1	-3.15	ns	1.77	-4.44	ns	2.21	1.28	2.85	-4.50	7.06	0.389	0.537	0.451	0.655

* *p* < 0.05; ** *p* < 0.01; ns not significant; 95% level of significance

## Data Availability

The datasets used and/or analyzed during the current study are available from the corresponding author.
